# The coupling and coordination degree and driving mechanism of water and land resources in the Beijing-Tianjin-Hebei urban agglomeration

**DOI:** 10.1371/journal.pone.0335717

**Published:** 2025-11-14

**Authors:** Weidong Chen, Meng Lian

**Affiliations:** Tianjin University School of Economics and Management, Tianjin, China; Federal University Otuoke, NIGERIA

## Abstract

Against the background of sustainable development, the use and development of water and land resources have become focal topics. In this study, the entropy method, Vector Autoregression (VAR), and the coupling degree model were employed to analyze the Beijing - Tianjin - Hebei (BTH) region as a case study. The reasons and driving mechanisms for the formation of low – coupling in cities were also analyzed. The results show that except for Shijiazhuang, the overall average coupling degree of the Beijing – Tianjin – Hebei urban agglomeration showed an upward trend (the coupling degree increased from 0.415 to 0.811 from 2013 to 2022), except for Shijiazhuang. Based on the principles of the driving mechanism, the following suggestions are proposed for Shijiazhuang City: Adjust the agricultural planting structure, select drought – resistant crop varieties, and reduce the planting area of high – water – consuming crops. Revise the “Regulations on Land and Water Conservation in Shijiazhuang City” and clarify the main body responsible for land and water conservation. In the agricultural field, it is necessary to promote non – full irrigation technology and improve the utilization efficiency of irrigation water.

## 1. Introduction

Water and land, as the two major fundamental and essential resources in nature, are key to maintaining human society, economic growth and ecological balance [1]. Water is the origin of all life, nourishing all organisms and driving the circulation of the Earth’s ecosystem; land, on the other hand, serves as foundation for human activities and development, playing multiple roles such as supporting agricultural planting, urban construction, and ecological habitation [2]. There is a close interdependence between water and land resources with the appropriate distribution and efficient use of water directly related to the fertility, productivity, and ecological stability of the land, and the rational use and protection of the land provide necessary support the preservation, purification, and sustainable use of water resources [3]. On one hand, the surge in population leads to increased food demand, the accelerated expansion of cities results in a greater demand for construction land, and the large consumption water resources by industrial development all contribute to the intensification of water and land resource development [4]. In order to meet the growing material needs, phenomena such as over-cultivation of, excessive extraction of groundwater, and unreasonable water resource management occur frequently, leading to increasingly serious problems such as land degradation and water shortage [5]. Heavy rain-induced erosion washes away nutrient-rich topsoil, disrupting land acidity and lowering agricultural productivity. The loss of vegetation cover also accelerates surface runoff, leading to river sedimentation and blockage. Consequently, flood risks rise, drought conditions may develop, and water scarcity intensifies for irrigation, industrial processes, and domestic use [6]. In some areas, the scarcity of water resources severely restricts agricultural irrigation, leading to a decrease in crop yields or even total crop failure, threatening security; industrial production faces the risk of shutdown or reduction due to a lack of water, affecting local economic development; and residents’ water use cannot be guaranteed, leading to a decline living quality [7].

Currently, the research directions of water and land resources can be divided into two broad categories. One focuses on the integrated study of water and resources, mainly analyzing the interactive relationship between these two elements and the impact of their changes on each other [8]. The other research tends to the basic theoretical field, starting from the perspective of degree, and analyzes water and land resources as two independent systems [9,10]. The integrated research direction emphasizes that water and land resources are closely connected in nature, and their healthy state directly affects ecological balance of the Earth [11]. In the research of the basic theoretical field, researchers focus more understanding the characteristics and rules of water and land resources [12]. They try to find scientific ways to optimize resource allocation and improve utilization efficiency.These studies provide important theoretical basis for the formulation of effective water and land resource management strategies [13].

Quantify the water and land resources in different regions, and applying these results to policy and resource management, are issues that need be addressed. The developing imbalance and chaos affect the efficient utilization and sustainable progress of resources [14]. Previous studies on coupling relationships but lacked coupling degree effects analysis, making it difficult to judge the system status and synergistic paths. In the process of system evolution, the development degree of sub-system that has been ignored affects the stability and function of the ecosystem [15,16].

Unlike previous studies, this article firstly incorporates the proportion of people using water and the supply capacity into the evaluation index, which not included in the evaluation system of previous research [17]. Traditional urban water security or water carrying capacity studies, which typically treat cities as isolated, homogeneous units, often rely on macro-indicators such as “total population” and “total water supply/use”. The limitation of this approach is that it obscures the enormous heterogeneity within urban agglomerations. This heterogeneity is a key factor that determines the challenges and priorities of coordinated development. The two indicators proposed in this study, “proportion of the population with access to piped water infrastructure” and “supply capacity based on actual infrastructure”, are innovative because they accurately characterize the core of water resource issues in urban agglomerations from an integrated “system-connected” and “socio-technical” perspective. By quantifying and analyzing the coupling degree of water and land resources in regions and different time scales, we expect to reveal the dynamic mechanism and evolution law behind it.At the same, this study will also focus on the development degree of the sub-system that has been ignored, explore its impact on the stability and function of the ecosystem, and provide new ideas methods for the sustainable utilization of water and land resources and the balanced development of the ecosystem [18–20].

## 2. Materials and methods

### 2.1. Study area

The B-T-H urban agglomeration is located in the central and northern part of North China Plain, covering the Haihe River and Luanhe River basins. According to the B-T-H land and Water Conservation Bullet (2023), the per capita water resources in this region are extremely low, such as only 191 cubic meters in Beijing, 160 cubic meters in Tianjin, and 386 cubic meters in Hebei, all of which are far below the national average. In 2023, the area of land erosion accounted for 18.90% of the total land area, with hydraulic erosion being the main type. Over the past decade, through comprehensive control and the construction of ecological – clean small watersheds, an additional 26,122.21 square kilometers of land has been governed, and 565 small watersheds have been built. The land is mostly brown land and yellow land, but there are problems of desertification and salinization, and 14.49% of the land in Hebei is degraded.

### 2.2. Establishment of the indicator system

The evaluation of water and land resources is a comprehensive and complex system, involving multiple fields. In order to clearly demonstrate the interaction between each component element in the WR(water resources)-LR(land resources)system system, this study developed a set of WR-LR evaluation index system. This paper adopts the per capita annual water supply to combine the total amount of water resources with the scale of population, eliminating the influence of population between regions and making the water resources conditions of cities, countries, or basins of different sizes comparable.

The per capita annual water supply volume directly reflects the water resource supply capacity of the regional water supply system, and can indicate whether a region has sufficient capacity to meet current water demand while addressing future challenges related to population growth and economic development. Water supply capacity directly determines the scale of urban development and the efficiency of land resource utilization, and becomes a core constraint factor for the land resource system through water carrying capacity.The amount of water per unit of arable land is a key indicator of water resources, as it directly reflects the matching relationship between arable land and water resources, serves as a core manifestation of agricultural water use efficiency, and forms a close connection with the land resource system through soil water holding capacity, water carrying capacity, etc. The population with access to clean water sources is a key indicator of water resources, as it is directly related to the balance between water supply and demand, reflects the carrying capacity of water resources, promotes social equity, and forms an indirect but close connection with urban development intensity and the land system.The per capita land area directly reflects the coordination between land resources and population development. The per capita arable land area indicates the per capita allocation of land resources and serves as a necessary parameter for measuring the carrying capacity of regional land resources. The grain yield per unit of arable land directly reflects the quality and production potential of land resources and is a key indicator of their sustainable utilization capacity. The per capita road area directly reflects the efficiency of land resource allocation, thereby revealing the intensive use of land resources [21–26].

In order to enhance the effectiveness of comparative analysis, we specifically used the per capita unit as the measurement benchmark. After comprehensive consideration of the particularity of the study area and the ease of data collection, we carefully selected 8 evaluation indicators with typical significance by referring to current scientific research achievements and the literature of resource integration (as shown in Table 1).

**Table 1:** Evaluation system of water and land resources.

Subsystem	Indicator	Direction
Water resources(WR)	Annual water supply per capita (Cubic meter/person)	+
	Water supply capacity (10,000 Cubic meter/day)	+
	Water consumption per unit of arable land (tonnes/Cubic meter)	–
	Population with access to water (%)	+
land resources(LR)	Land area per capita (square kilometre/10,000 people)	+
	Area of arable land per capita (square kilometre/10,000 people)	+
	Grain yield per unit of arable land (tonnes/square kilometre)	+
	Road area per capita (square meter)	+

When normalizing based on quantity, preprocess the ‘positive indicators’ and ‘negative indicators’ separately [27]. This study standardized the urban datasets and imputed missing values for individual cities using linear interpolation.

Processing of positive indicators:


$$Z_{ij}=\frac{X_{ij}-X_{\min}}{X_{\max}-X_{\min}}$$ (1)

Processing of negative indicators:


$$Z_{ij}=\frac{X_{\max}-X_{ij}}{X_{\max}-X_{\min}}$$ (2)

Among them, $X_{ij}$ and $Z_{ij}$ They refer to the original data and the standardized data of indicator j in year i, respectively.

Calculate the entropy value of the Jth indicator.


$$e_{j}=-k\sum_{i=1}^{n}p_{ij}\ln\;(p_{ij}),j=1,\cdots ,m$$ (3)

Among them, k=1/ln (n)>0 satisfy $e_{ij}\geq 0$

Calculate the redundancy of information entropy (difference):


$$d_{j}=1-e_{j},j=1,\cdots ,m$$ (4)

Calculate the weights of various indicators:


$$w_{j}=\frac{d_{j}}{\sum_{j=1}^{m}d_{j}},j=1,\cdots ,m$$ (5)

Calculate the comprehensive score:


$$S_{i}=\sum_{j=1}^{m}w_{j}x_{ij},i=1,\cdots ,n$$ (6)

Among them, $X_{ij}$ represents the normalized data. The factors can be ranked based on the scores of the factors.

### 2.3. Coupling coordination degree

Coupling degree, originating from the physical sciences, is now widely used to measure the closeness of interaction among complex systems in social sciences, economies, the environment, and other fields [28]. In the context of regional sustainable development and resource optimization allocation, the concept of coupling degree is particularly important [29,30]. The specific construction steps are as follows:


$$u_{1}=\sum_{i=1}^{m}w_{ij}u_{ij}$$

$$\sum_{j=1}^{m}w_{ij}=1$$

$u_{i}$ is the contribution of subsystem i to the entropy of the total system; $u_{ij}$ is the normalized value of indicator j in subsystem i; $w_{ij}$ the weight of indicator j in subsystem i, which is calculated by the entropy weight method.

The entropy weight of the operator system needs to be normalized before it is used, using the maximum-minimum method: the larger the $u_{ij}$, the (positive).


$$u_{ij}=\frac{x_{ij}-x_{\min}}{x_{\max}-x_{\min}}$$

$u_{ij}$ for when a smaller value is better for the system (negative)


$$u_{ij}=\frac{x_{\max}-x_{ij}}{x_{\max}-x_{\min}}$$

Due to the difficulty of fully reflecting the effectiveness and synergy of subsystems, and the dynamic imbalance of extreme values, it is easy to mis, so coupling coordination is proposed.


$$C=2{\left\{\frac{\left(u_{1}\cdot u_{2}\right)}{{\left(u_{1}+u_{2}\right)}^{2}}\right\}}^{1/2}$$

$$D=(C\cdot T{)}^{1/2}$$

$$T=au_{1}+bu_{2}$$

where C is the coupling degree, D is the coupling coordination degree, $u_{1}$ and $u_{2}$ denote the index of water resources and land resources respectively, a and b refer to the weights of the index of water resources and land resources (The weights of a and b were determined through linear weighting, following data standardization and the entropy method).

A high D value indicates a high degree of coordination among subsystems. According to the standards set by predecessors, it is divided into four levels: high quality, barely coordinated, uncoordinated, and seriously uncoordinated [31]. Each level is further subdivided [32–36], and the coordination level is visualized through color labels [37–40].As shown in Table 2.

**Table 2:** Standards for system coupling coordination evaluation levels.

Coupling Level	C (coupling degree)	Coordination level	D (coordination index)	Coordinating subgrade
High level of coupling	0.8 < C ≤ 1.0	High-quality coordination	0.9 < D ≤ 1.0	High-quality coordination
			0.8 < D ≤ 0.9	well coordinated
Adaptation stage	0.5 < C ≤ 0.8	Barely coordinated	0.7 < D ≤ 0.8	Intermediate coordination
			0.6 < D ≤ 0.7	Entry-level coordination
			0.5 < D ≤ 0.6	Barely coordinated
Antagonistic phase	0.3 < C ≤ 0.5	Slight dyscoordination	0.4 < D ≤ 0.5	On the brink of imbalance
			0.3 < D ≤ 0.4	Mild dysregulation
Low-level coupling	0.0 < C ≤ 0.3	Serious disharmony	0.2 < D ≤ 0.3	Moderate dysregulation
			0.1 < D ≤ 0.2	Serious imbalance
			0.0 ≤ D ≤ 0.1	Extreme dysfunction

### 2.4. VAR vector auto-regressive model

The VAR model is used to assess the dynamic interaction among multiple variables. Building separate autoregressive models for X1 and X2 allows us to evaluate their dynamic relationship. However, this approach may not fully account for potential endogeneity. In contrast, simultaneous equations modeling explicitly captures their mutual interdependence, providing a more comprehensive framework for analysis.For example, when there are multiple variables in a system, the VAR model treats each variable as the dependent variable Y and uses the lagged values of all variables in the system as independent variables to construct equations. Therefore, the number of variables in the system determines the number of equations that can be established, and these equations are able to depict the dynamic relationship between variables [41–43].

The formula is as follows:


$$Y_{t}=\mu +\Pi_{1}Y_{t-1}+\Pi_{2}Y_{t-2}+\cdots \Pi_{t}Y_{t-k}+u_{t},u_{t}\sim \Pi \Pi 0,\Omega$$

Among them,


$$Y_{t}=(y_{1},y_{2},t\cdots y_{N},t)$$

$$\mu =(\mu_{1},\mu_{2}\cdots \mu_{N})$$

$$u_{t}=(u_{1t},u_{2t},\cdots ,u_{Nt})$$

$$\Pi_{j}=\left[\begin{array}{cccc} {}*20c\pi_{11.j} & \pi_{12.j} & \cdots & \pi_{1.N.j}\\ \pi_{21.j} & \pi_{22.j} & \cdots & \pi_{2.N.j}\\ \vdots & \vdots & \ddots & \vdots \\ \pi_{N1.j} & \pi_{N2.j} & \cdots & \pi_{NN.J} \end{array}\right],j=1,2,\cdots ,k$$ (7)

With two variables $y_{1t}$,$y_{2t}$ Using a VAR model with a lag of 1 as an example:


$$\left\{ \begin{array}{c} y_{1,t}=\mu_{1}+\pi_{11.1}y_{1,t-1}+\pi_{12.1}y_{2,t-1}+\mu_{1t}\\ y_{2,t}=\mu_{2}+\pi_{21.1}y_{1,t-1}+\pi_{22.1}y_{2,t-1}+\mu_{2t} \end{array}\right.$$ (8)

Among them, $(u_{1t},u_{2t}\sim \Pi \Pi D0,\sigma^{2}),Cov(u_{1t},u_{2t})=0$

In matrix form, it is represented as:


$$\left[\begin{array}{c} y_{1t}\\ y_{2t} \end{array}\right]=\left[\begin{array}{c} \mu_{1}\\ \mu_{2} \end{array}\right]+\left[\begin{array}{cc} {}*20c\begin{array}{c} \pi_{11.1}\\ \pi_{21.1} \end{array} & \begin{array}{c} \pi_{12.1}\\ \pi_{22.1} \end{array} \end{array}\right]\left[\begin{array}{c} y_{1,t-1}\\ y_{2,t-1} \end{array}\right]+\left[\begin{array}{c} u_{1t}\\ u_{2t} \end{array}\right]$$ (9)

## 3. Results

### 3.1. WR-LR coupling degree

**Fig 1:**
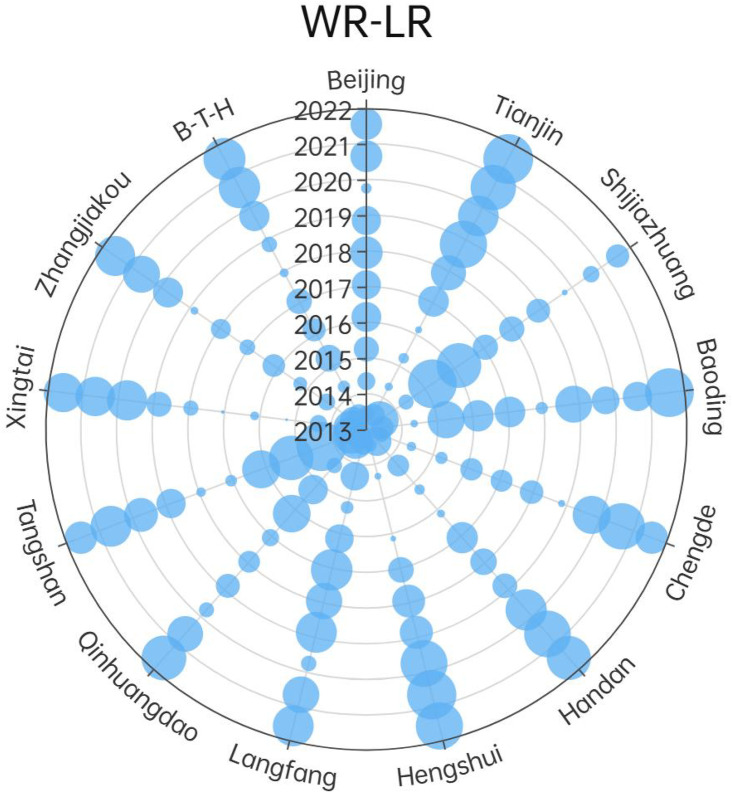
Coupling degree results of WR-LR.

Observing the data in the Fig 1, it is evident that the coupling degree difference between urban agglomerations is significant. Taking the two major metropolises of Beijing and Tianjin as examples, their coupling degrees are generally high, which indicates their relative coordination in the management of water and land resources. Relatively, smaller urban agglomerations like Chengde and Zhangjiakou have significant fluctuations in coupling degrees, indicating that these regions may face more challenges in the use and management of water and land resources. Besides, from the time series analysis, although the coupling degree fluctuates overall, it has shown an upward trend in recent years, mainly due to the improvement and implementation of water and land resource policies. Due to the lack of an effective coordination mechanism, there are some unreasonable aspects in the protection, which exacerbates the problem of low coupling degree. Due to the implementation of large-scale water and land conservation projects, the coupling degree of this area has increased after 2019.

which have also promoted the ecological environment improvement of the region and enhanced the carrying capacity of regional water resources. By promoting water-saving irrigation, rainwater collection and utilization, and other agricultural water-saving technologies, the utilization efficiency of water resources has been improved. At the same time, strengthening the supervision of industrial and domestic water use, and promoting water-saving appliances and water systems, has further reduced the consumption of non-agricultural water use.

Observing at the urban level, during the period from 2013 to 2016, except for Shijiazhuang, Baoding, Cangzhou, Langfang, Qinhuangdao, Tangshan, and Xingtai, other cities all exhibited a state of low coupling degree. The coupling degree of Shijiazhuang decreased after 2016, mainly due to the pollution of surface water bodies such as rivers and lakes by industrial wastewater and domestic sewage, with varying degrees of pollution. The development trend of other cities is similar to the overall trend of the Beijing-Tianjin-Hebei region, which is the transformation from a low-coupling state to a high-coupling state. This change is attributed to a series of policies implemented in the Beijing-Tianjin-Hebei region, which aim to protect water and land resources, carry out ecological restoration, and utilize resources rationally. For example, large-scale afforestation and greening projects are vigorously carried out, water-saving technologies are promoted, and the management and protection of groundwater are strengthened. The exploitation of groundwater is strictly limited, and the recharge and replenishment of groundwater are strengthened.

### 3.2. VAR model analysis

#### 3.2.1. Lag order selection.

To ensure the overall validity of the impulse response and variance decomposition analyses, the optimal lag order was selected based on computational results, ultimately determined to be one. Detailed results are presented in Table 3, both WR and LR passed the test at the 1nd order of differencing, indicating that the variables are stable and that the 1nd order differencing is the optimal order.

**Table 3:** Results of the lag order selection calculation.

lag order	AIC	BIC	HQIC
0	−8.488	−8.428	−8.554
1	−9.879*	−9.748*	−10.163*
2	−9.063	−8.964	−9.733

**Fig 2:**
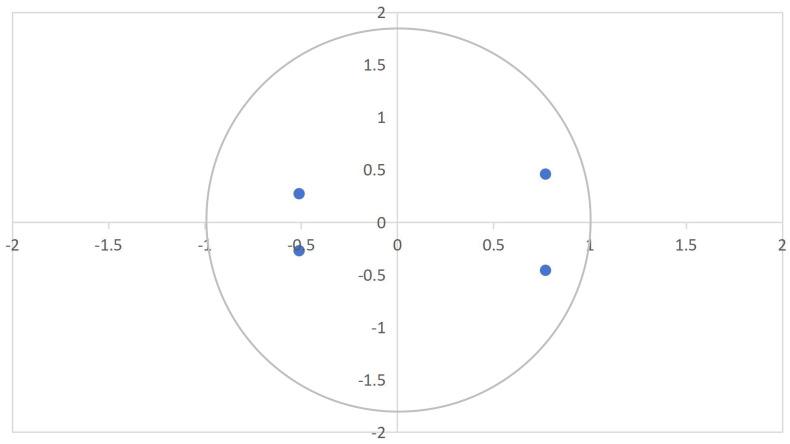
Stability inspection results. This figure displays the autoregressive (AR) root diagram of the VAR model. Stability is confirmed if all roots lie inside the unit circle, enabling further impulse response analysis.

Based on the ADF test results, the stability of the model has been confirmed, as specifically illustrated in Fig 2.

#### 3.2.2. Impulse response analysis.

**Fig 3:**
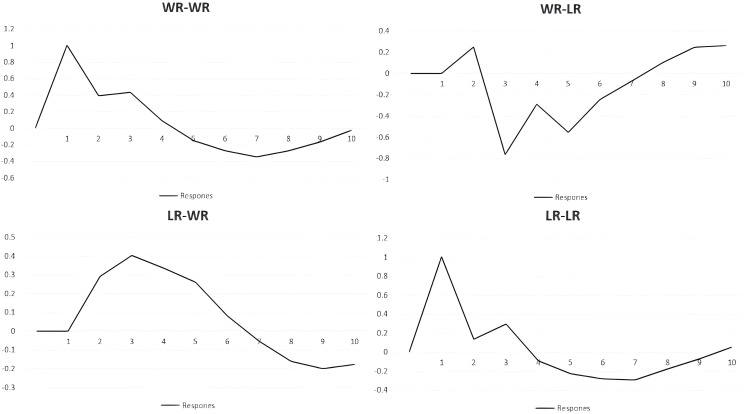
Pulse response diagram. Note: In the figure, the X-axis represents the order of the pulses, and the Y-axis represents the response values. Specifically, WR-WR denotes the impulse response function between water resources and water resources, WR-LR represents that between water resources and land resources, LR-WR indicates the function between land resources and water resources, and LR-LR signifies the impulse response between land resources and land resources.

From Fig 3, we observe a time lag in the interaction between water resources (WR) and land resources (LR). The impulse response function illustrates how a shock to one variable affects the other over time, including immediate and lagged responses, as well as positive/negative fluctuations.

When WR is shocked:WR’s effect on itself is initially positive but turns negative after the fourth lag period, with a significant rebound (reaching −0.029) between the seventh and tenth lags.WR’s shock on LR is positive during the first two lags but becomes negative from the third to seventh lags.

When LR is shocked:LR’s impact on both itself and WR follows an inverted U-shape.For WR, LR’s shock initially has a positive but weakening effect, with the negative phase narrowing after the ninth lag.For LR itself, the shock effect rises initially, peaks, then declines; the negative effect diminishes by the seventh lag and turns positive after the tenth lag.

#### 3.2.3. Decomposition of variance results.

Variance decomposition reveals the dynamic relationships between variables by calculating the contribution of these shocks to the forecast error variance of future values of endogenous variables.

As shown in the Table 4 all variables exhibit independence characteristics in the short term (lag1). Water resource fluctuations are entirely driven by themselves (WR contributes 100% to itself, while LR contributes 0%), and land resource fluctuations are highly dependent on themselves (LR contributes 99.702% to itself, while WR contributes 0.298%).

**Table 4:** Decomposition of variance results.

Variables	lag	WR(%)	LR(%)
WR	1	100	0
	5	48.246	51.754
	10	47.563	52.347
LR	1	0.298	99.702
	5	21.5	78.5
	10	21.516	78.484

In the medium term (lag5), mutual influence strengthens. For water resource fluctuations, the contribution of land resources surges to 51.754%, while for land resource fluctuations, the contribution of water resources increases to 21.5%.

In the long term (lag10), a bidirectional dominant relationship emerges. For water resource fluctuations, the contribution of land resources stabilizes at over 52%, while for land resource fluctuations, the contribution of water resources stabilizes at around 21.5%. Additionally, it can be observed that the contribution ratios of both tend to stabilize in the long term, with a certain degree of interaction. Notably, the influence intensity of land resources on water resources (51.754% in the medium term, 52.347% in the long term) is significantly higher than that of water resources on land resources (21.5% in the medium term, 21.516% in the long term), indicating that land resources are the dominant external factor driving changes in water resources.

### 3.3. Analyze the driving mechanism of the development of water and land resources (WR-LR)

**Fig 4:**
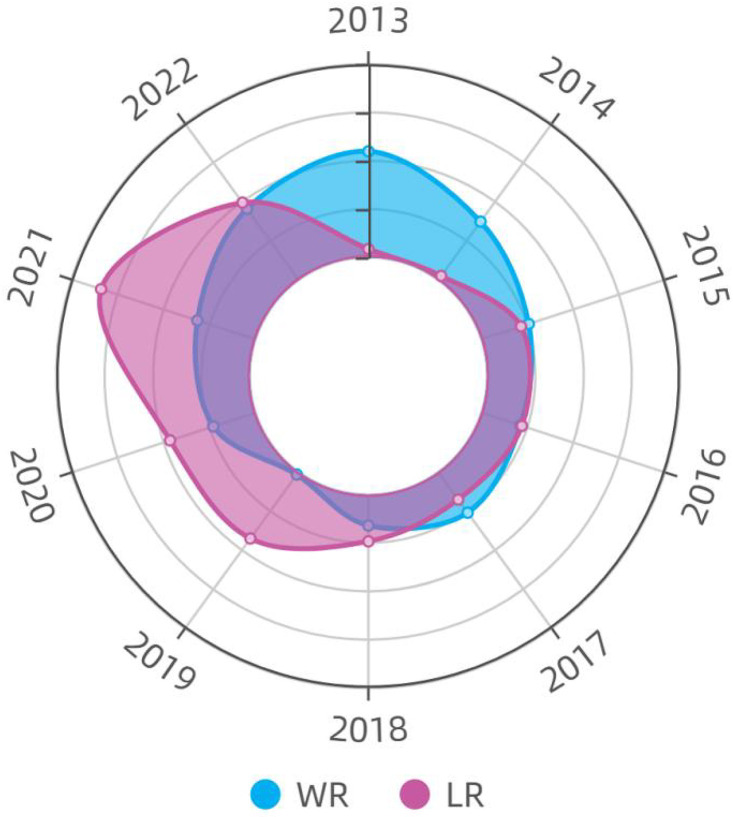
WR-LR driving mechanism.

Through Fig 4, from an overall perspective, it can be seen that the driving force mainly depends on LR. This is mainly due to the fact that the industrialization and urbanization of the region are developing simultaneously, and the continuous gathering of population and industries to cities and surrounding areas. In this process, land resources have been utilized more efficiently, and the difference in land resource utilization among different regions has gradually narrowed. Especially in some counties around Beijing and Tianjin, land resources have developed due to the radiation diffusion effect of developed areas, and these regions have become areas with a high degree of development of population, land, and industrial elements.

However, the uneven distribution of precipitation in this area, coupled with the pollution and waste of water resources, has led to a prominent water shortage problem in some parts, also limits the driving force for water resources development.For example, some areas still use traditional flood irrigation, resulting in a large amount of water resource waste. The second issue is the distribution problem: precipitation in this region is highly concentrated in summer, especially in July and August, with persistent drought in winter. This phenomenon of uneven seasonal distribution of precipitation exacerbates the tension of water resources.In areas with water shortage, due to the lack of water supply, agricultural irrigation is restricted, and industrial development is also restricted, affecting the local economic and social development. In addition, water shortage may also lead to the deterioration of the environment, such as the drying up of lakes and rivers and the reduction of wetlands, which further exacerbates the differences between regions.

## 4. Discussion

The evaluation of relevant indicators in existing research on water-land coupling development lacks a unified standard, and the research methods are overly homogeneous. To more accurately reveal the dynamic correlation and complex interaction mechanism within the system, this study innovatively introduces panel data based on time series and constructs a VAR model. The application of this model provides a powerful tool not only for understanding the dynamic changes within the water-land resource system but also for revealing the complex and subtle interaction relationship between these systems. The results of the study show that some cities, such as Beijing and Tianjin, face more severe water-land resource pressure due to their highly concentrated economic activities and large population base. In contrast, some cities in Hebei may have a relatively relaxed situation in the utilization and management of water-land resources.

A key distinction of this study is that groundwater resources are not included as an evaluation index. This decision stems from two factors: first, the coastal cities in the study area face a high risk of seawater intrusion due to excessive groundwater exploitation, as evidenced by similar incidents in Shanghai; second, the distribution of rivers and reservoirs in the B-T-H region is highly uneven, with some cities having abundant water resources while others lack inland rivers entirely. Therefore, this study tailors its evaluation framework to local conditions, formulating indices that reflect the actual hydrogeological characteristics of the region, rather than arbitrarily excluding water-related metrics. This approach represents an innovation compared to previous research.

The VAR model results indicate a complex lagged interaction between WR and land LR, revealing the long-term leverage effect of land-use planning on water resource health.Shocks in land-use planning exert a persistent impact on water resources—for example, cropland expansion or increased urban impervious areas alter surface runoff patterns and reduce soil infiltration capacity, leading to sharp declines or even complete interruption of river flow, while simultaneously aggravating non-point source pollution and deteriorating water quality.There is also a feedback regulatory demand from water resource policies on land resource utilization. Policies such as water resource tax reforms and the operation of the central route of the South-to-North Water Diversion Project alter the cost and supply structure of water resources. In the short term, these changes may trigger adjustment challenges in land-use practices, such as increased water costs for enterprises leading to constrained production and affecting land development intensity.

In addition, this study also notes that with the continuous advancement of urbanization, the coupling relationship of water-land resources may face new challenges, such as the occupation of cultivated land by urban expansion and water pollution due to over-exploitation, which require our continuous attention and the taking of corresponding measures. Although the current academic literature on the coupling degree of resources is quite rich, there are still some gaps and challenges in exploring the interaction mechanism between subsystems and constructing a unified and comprehensive evaluation system. In view of this, this study takes a novel research path to deeply explore the interaction mechanism between water and land resources. Different from the traditional evaluation methods, this study starts from a unique statistical perspective and carries out a comprehensive, systematic measurement and scientific evaluation of water-land resources.

## 5. Suggestions

In order to improve the coupling degree of water and land resources in the B-T-H region in the process of development, we propose the following suggestions:

Given the current situation where LR development intensity lags behind WR availability in cities such as Baoding, Chengde, Cangzhou, Hengshui, Tangshan, Xingtai, and Zhangjiakou, differentiated policies based on the principle of “water-determined land use with intensive efficiency” must be formulated.In water-scarce cities like Cangzhou and Tangshan, expansion of high water-consumption industries should be strictly restricted, with priority given to land use for water-efficient industries. A “Red-Yellow-Blue” zoning control mechanism based on water resources should be established:Red zones(overloaded areas) prohibit new construction land;Yellow zones(near-overload areas) restrict land development intensity;Blue zones(sustainable areas) allow moderate expansion of development.In cities with relatively abundant water resources such as Chengde and Zhangjiakou, a joint mechanism integrating water rights trading and land development should be promoted. A water rights trading market could be explored, allowing enterprises to exchange purchased water rights for land development quotas, thereby directing water resources toward high-value uses. Enterprises with advanced water-saving technologies and high water recycling rates should receive policy incentives such as prioritized land supply and floor area ratio rewards.Baoding and Hengshui, as major agricultural regions, should consolidate fragmented farmland into concentrated, high-standard fields through land remediation, supported by water-saving irrigation facilities to reduce water consumption per unit of farmland. In combination with saline-alkali land improvement, drought-resistant crop cultivation should be promoted to lower agricultural water demand.Xingtai, as an industrial city, could implement a “standard land transfer” system, specifying requirements for investment intensity, output value, and tax revenue per unit of land to incentivize enterprises to improve land use efficiency. Enterprises undertaking multi-story factory construction or developing underground space should receive floor area ratio incentives and tax reductions.

Given the current situation where land resource development intensity exceeds water resource availability in cities such as Beijing, Tianjin, Langfang, Handan, and Qinhuangdao, differentiated policy frameworks must be formulated based on each city’s land use characteristics and development needs. Beijing should strengthen the protection of water sources such as the Miyun and Guanting Reservoirs, designate permanent ecological water replenishment channels, and ensure a stable annual urban water supply of over 2.5 billion cubic meters. Groundwater over-exploitation zones should be subject to “negative growth” management, with groundwater levels restored by 2030. Strict implementation of the Comprehensive Land Consolidation Implementation Guidelinesshould be enforced, focusing on consolidating fragmented farmland in suburban plains and reclaiming inefficient construction land through the linkage between urban and rural construction land use.Tianjin must strictly enforce quota management for water diverted from the Luanhe River and the Central Route of the South-to-North Water Diversion Project, while establishing a cross-regional water resource compensation mechanism. Ecological flow guarantees should be implemented for the mainstream of the Haihe River and its tributaries, such as the Ziya River and Duliujian River, to ensure basic ecological water use during dry seasons. The city should fully implement the “Measures for Promoting High-Quality Land Resource Utilization in Tianjin,”applying “zero-growth” controls in areas where development intensity exceeds 30%, such as Binhai New Area and Xiqing District. The allocation of new construction land should be linked to the disposal of existing unused land, and the rate of idle land disposal should be included in district-level performance evaluations. Langfang could develop ecological flood detention zones in low-lying areas such as Wen’anwa and Dachengwa, enhancing stormwater storage capacity through wetland restoration to reduce reliance on groundwater. In accordance with the Interim Measures for the Preparation of Comprehensive Implementation Plans for Inefficient Land Redevelopment, industrial parks in Wen’an County and Bazhou City should undergo “industrial upgrading,” phasing out high water-consuming, low-output enterprises and introducing low water-consuming industries such as biomedicine. Handan should seal 70% of the polluted shallow groundwater in the eastern plains, promote industrial water recycling technologies in enterprises like Hansteel and Ma’elec Power, and raise the industrial water reuse rate to over 90%. In major agricultural counties such as Jize and Yongnian, a “land consolidation + industrial integration” model should be adopted, adding 100,000 mu of farmland through comprehensive land consolidation while simultaneously establishing agricultural product processing zones.Qinhuangdao should leverage coastal wetlands to build a “sponge city” ecological barrier, implement ecological restoration for rivers like the Daihe and Yanghe flowing into the sea, and restore natural river shorelines. “Point-based land supply” should be applied in tourist functional areas like Beidaihe New District and Shanhaiguan District, allowing tourism facility land to be allocated in phases based on actual demand to prevent land idling.

For Shijiazhuang, it is imperative to treat water resource carrying capacity as the core constraint for land development. In groundwater over-exploitation zones such as Gaocheng District, prohibited and restricted extraction areas should be delineated, with mandatory closure of illegal motorized wells by 2025. Ecological water replenishment should be carried out through rivers such as the Hutuo River and Ziya River, while water resources should be substituted via the South-to-North Water Diversion Project to achieve a balance between extraction and recharge. Additionally, reclaimed water must be formally integrated into unified water resource allocation, with priority given to its use in industrial cooling and urban greening.

Land reserves should be prioritized in areas with abundant water resources, while expansion of land for high water-consumption industries must be restricted. When reserving land along the Hutuo River, water-efficient industrial projects should be given precedence. In older industrial zones such as Xinhua District and Qiaoxi District, an “industrial upgrading” strategy should be implemented to phase out high water-consuming enterprises and enhance land use efficiency through three-dimensional development and mixed-use approaches. Furthermore, the construction of an “underground logistics corridor” in Chang’an District could relocate storage and distribution functions below ground, reducing surface land occupation and lowering water consumption associated with logistics transportation.

Industrial land supply flexibility should be introduced: High-tech industrial development zones may adopt a “lease before transfer” model for industrial land, whereby enterprises must meet water-saving standards to qualify for land use extension.

## 6. Conclusions

The main conclusion of the study, based on an analysis of the findings, is as follows: During the period from 2013 to 2022, excluding Shijiazhuang City, the B-T-H urban agglomeration has demonstrated an overall positive growth trend. This indicates that, with the progress of the economy and society, as well as the promotion of relevant policies. The analysis of coupling degree and linear weighted values indicates two distinct scenarios: 1. In Baoding, Chengde, Cangzhou, Hengshui, Tangshan, Xingtai, and Zhangjiakou, land resource development is less intensive than water resource development, suggesting a need to strengthen land resource utilization policies. 2. For the remaining cities, land resource development exceeds water resource development, emphasizing the urgency of enhancing water resource conservation and infrastructure. This finding implies that for the sustainability of the region, land resource management must become the core focus of ecological conservation and economic development, while systematic policy design is needed to balance short-term demands and long-term carrying capacity in resource utilization.

First, the manner of land resource utilization directly determines the threshold of water resource carrying capacity. The root cause of water scarcity in the Beijing-Tianjin-Hebei region lies in the mismatch between land use structure and water resource distribution. For instance, an excessively high proportion of agricultural land leads to a surge in irrigation water demand, while increased impervious surfaces due to urban expansion reduce rainwater infiltration, further exacerbating groundwater over-extraction. If land resource management lacks forward-looking planning—such as continuing to expand the cultivation of high-water-consumption crops or promoting disordered urbanization—it will directly exceed the water resource carrying capacity threshold, increasing the risk of ecosystem collapse.

Second, there is currently a disconnection between land and water resource policies in the Beijing-Tianjin-Hebei region. Land use planning often fails to adequately account for water constraints, resulting in land development in some areas exceeding local water carrying capacity. Meanwhile, water management policies have not effectively guided adjustments in land use practices—for example, water pricing reforms have failed to incentivize the phase-out of high-water-consumption industries. If land resources are identified as the core driver of water-land interactions, policies must establish a coordinated “water-determined land use” mechanism, making water resource carrying capacity a precondition for land use planning.

This study provides information support for establishing an ecological compensation fund with a dynamic adjustment mechanism, where the fund scale is linked to core indicators such as water quality, water quantity, and forest coverage. By leveraging the quantitative findings of this research, it offers robust evidence for formulating specific plans in line with the national Regulations on Ecological Protection Compensation, covering compensation principles, subject rights and responsibilities, standard calculation methods, fund management protocols, and supervision and accountability frameworks.

However, as a special case, the development status of the coupling and coordination of water and land resources in Shijiazhuang City warrants special attention. Shijiazhuang once faced the crisis of “all rivers drying up and all water bodies being polluted,” exemplified by incidents such as the Mian River blackwater event and reservoir pollution in Luquan District. In the initial phase of pollution control, measures like shutting down polluting enterprises and restricting irrigation of farmland along the banks weakened the traditional dependency between land and water resources in the short term. For instance, during the early stage of ecological restoration of the Hutuo River, reducing agricultural irrigation water was necessary to improve water quality, leading to a temporary mismatch between the intensity of land resource development and the efficiency of water resource utilization.

After the central route of the South-to-North Water Diversion Project began supplying water, the surface water availability in Shijiazhuang increased, effectively alleviating water scarcity. However, in the initial phase of ecological water supplementation, the recovery of river ecological functions required time—for example, the Hutuo River achieved full restoration of flow only in 2021. During this stage, a time lag existed between the increase in water resource availability and the improvement of the ecological carrying capacity of land resources, resulting in a short-term decline in the coupling degree.

Therefore, in the future development, Shijiazhuang City needs to pay more attention to the protection and rational utilization of water and land resources, reduce the proportion of heavy industry through industrial structure adjustment and upgrading, improve the level of clean production and resource recycling, so as to achieve the coordinated development of economy and environment. At the same time, the government should also increase its investment and support for the protection of water and land resources in Shijiazhuang City, and promote the in-depth development of water and land conservation in this region.

## Supporting information

S1 Data Original data. (XLSX)
